# A “transmission gap” between research and practice? A Q-methodology study of perceptions of the application of attachment theory among clinicians working with children and among attachment researchers

**DOI:** 10.1080/14616734.2022.2144393

**Published:** 2022-11-17

**Authors:** Helen Beckwith, Marinus van IJzendoorn, Mark Freeston, Matt Woolgar, Paul Stenner, Robbie Duschinsky

**Affiliations:** a Department of Public Health and Primary Care, University of Cambridge, UK; b Cambridgeshire and Peterborough NHS Foundation Trust, UK; c Psychoanalysis Unit, University College London, UK; d Erasmus University Rotterdam, Netherlands; e School of Psychology, Newcastle University, UK; f Institute of Psychiatry, Kings College London, UK; g South London and Maudsley NHS Foundation Trust, UK; h Faculty of Arts & Social Sciences, Open University, UK

**Keywords:** Attachment theory, clinical practice, Q-methodology, Diagnosis, Scientific knowledge

## Abstract

Clinical practitioners are frequently encouraged, through literature, training, and policy, to learn, understand, refer to and use their knowledge of attachment theory and research when working to meet the needs of children and families. However, there has been very little empirical study of how practitioners understand and perceive the relevance of attachment concepts and methods. Q-methodology was used to examine the perceptions of attachment knowledge and its applications for practice among 30 UK clinicians working with children and an international group of 31 attachment researchers. Factor analysis revealed three perspectives, described as: i) pragmatic, developmental, and uncertain, ii) academic, and iii) autodidactic and therapeutic. Participants agreed on core tenants of theory, their aspirations for clinical practice and the inaccessibility of current assessment measures for practitioners. Yet they diverged on their understandings of attachment insecurity, disorganisation, and the implications of both for various aspects of child development.

## Introduction

There are an extraordinary number of books and articles advising practitioners on how to use attachment theory in different areas of practice. One area of application has been attachment-based interventions across the lifespan, and their evaluation (Steele & Steele, 2018). Yet besides process evaluations of attachment-based interventions (e.g. Stolk et al., 2008) and a recent survey of attachment classification use in Sweden (Hammarland, et al., 2022), there has been almost no research evaluating how current clinical or welfare practice has actually been shaped by this substantial body of literature. There is a particular paucity of empirical knowledge surrounding practitioners’ understanding of attachment concepts and their relevance.

The lack of available research appraising current practice has not deterred a wealth of recommendations and guidelines being published and circulated. The most evidenced-based examples include diagnostic recommendations from large-scale research, such as the ALSPAC outputs (Chaffin et al. 2006) and the updated practice parameters for use with DSM-5 criteria (Zeanah et al., 2016). Other examples of attachment-related literature aimed at clinicians include topics of children with developmental trauma (Crittenden, 2016; Hughes, 2007), psychotherapy with adults (Holmes & Slade, 2017; Johnson, 2019; Wallin, 2007), family and systemic therapy (Crittenden et al., 2014; Dallos & Vetere, 2009), service design and delivery (Bucci et al., 2015; Goodwin, 2003), education (Geddes, 2006; Golding et al., 2012), child welfare assessments (Howe et al., 1999; Shemmings & Shemmings, 2014) and family court work (Hontree, 2017), to name a few.

Some recommendations for practitioners (e.g. Forslund et al., 2021) are comparatively well grounded in the available empirical evidence. However, even within otherwise well-conceived guidelines, gaps in knowledge of how attachment theory and research may relate to applied practice have led to ill-judged proposals. To give one example, the team of experts recruited by the UK’s National Institute for Clinical Excellence (NICE) to develop the national guidelines for attachment (NG26, (National Institute for Health and Care Excellence (NICE), 2015) briefly considered implementing routine assessments of infant attachment as part of a national screening programme (see consultation document, National Collaborating Centre for Mental Health, 2015b). The scope of this guidance was intended for children in, adopted from or at risk of going into care. However, the proposition of national screening demonstrated the potential of attachment concepts to be applied so widely as to include public health considerations of all children born in the UK. This was considered without adequate understanding of population vs individual level validity of the assessment tools and was halted by researchers and professional bodies appealing to the NICE guideline committee in feedback on the first draft (Clinical guidelines for consultation, May 2015, p79–81).

Yet clinicians have raised concerns about inappropriate assessment (Allen & Schuengel, 2020; Woolgar & Baldock, 2015; Woolgar & Scott, 2015) and treatment decisions being made in the name of attachment theory (see Prior & Glaser, 2006; Zeanah et al., 2016). Other scholars have acknowledged difficulty in maintaining the integrity of attachment concepts and methods when applying them meaningfully to practice (e.g. Oppenheim & Goldsmith, 2007). A recent illustration was the training of over 5,000 social workers in the UK to use identification of disorganised attachment in naturalistic settings as an indicator of child maltreatment (Shemmings & Shemmings, 2014; see also Wilkins, 2021). Responding to such misapplications of attachment theory and research, attachment scholars have undertaken international consensus statements on key matters of disorganised attachment (Granqvist et al., 2017) and child protection and custody issues (Forslund et al., 2021). However, these consensus statements have expressed concern about the lack of empirical evidence regarding actual practitioner knowledge on which to draw and emphasise that these works of consensus were premised only on anecdotal information and speculation regarding professional understanding and practice. Both consensus statements highlighted the importance of research on professional understanding of attachment theory and research, and empirical specification of lines of alignment and misalignment between the perspectives of researchers and practitioners.

In a historical analysis, Duschinsky (2020) has claimed misalignment between researchers and practitioners is worse in attachment research than in other areas of psychology; a concern echoed by White and colleagues (2020). However, recognising that their analysis was limited by a focus on document analysis, both have called for empirical research to examine the perspectives of professionals. Earlier this year, Hammarlund et al. (2022) reported survey findings from a nationally representative sample of Swedish child protection workers. They also concluded that there is a wide researcher-practitioner gap, noting a significant lack of validated assessment methods in practice combined with an overconfidence in perceived implications of attachment classifications. Prior to this, the most direct attention to the issue was a study by Wilkins (2016) who found that ideas of attachment appealed to social work practitioners because they emphasised the importance of relational issues, emotion, the impact of early experience on anxiety and the experience of symptoms. This was part of the spur for Wilkins’ enthusiasm for the use of attachment assessments in child protection practice, a position he has subsequently retracted following the international consensus statement (Wilkins, 2020). Beyond this, two interview studies with social workers reported that participating professionals had insufficient knowledge of attachment theory and interventions: reporting the use of a “common sense approach” which was found to have little coherence with underpinning theory (Botes & Ryke, 2011) or non-standardised observations and assessments due to a lack of skills and resources (Lesch et al., 2013). One attempt to interview residential childcare staff found participants lacked an explicit awareness of attachment theory and thus had difficulty articulating theory-practice links (Morison et al., 2020). Instead, they described their practice as “natural” and “common sense,” focused on “building relationships” with “theory in the background” (p.9).

The available literature remains scant on practitioner understandings of attachment, with all empirical enquiries so far focused on social welfare practice and none on clinical contexts. In the consensus statements on disorganised attachment and attachment in court practice, as well as in the work of some academic commentators, it is sometimes assumed that there is a “transmission gap” in knowledge between researchers and practitioners, where the latter lack knowledge held by the former. No doubt this is true in certain regards, yet critics have challenged the knowledge-translation metaphor for professional healthcare practice (Greenhalgh & Wieringa, 2011). As it stands, we do not know what understandings clinicians hold and where these converge or diverge with those of researchers and believe that a more sophisticated inquiry of both is needed. Additionally, we hope that greater specification of clinician understanding will highlight and elevate the status of practical wisdom and tacit knowledge, sometimes described as clinical “mindlines” (guidelines-in-the-head, Gabbay & le May, 2011) in a healthcare context. Although we are aware that attachment theory and research may feature in clinical practice across the lifespan, an enquiry focused on clinical work with children and families in the UK is a sensible first step given the specific encouragement from national guidance to work with an awareness of children’s attachment across all areas of child health (NICE, 2015; NG26).

### The present study

Q-methodology has been used effectively for the related purpose of identifying how child protection social workers use attachment theory with children who have been abused or neglected and identified four distinct perspectives on such conduct (Wilkins, 2016). It is a helpful approach for unearthing perspectives without requiring participants to articulate these clearly. This is advantageous to our enquiry as the existing interview-based studies revealed staff have observable difficulties with explicitly discussing both theory-practice links and attachment specifically (Morison et al., 2020). Indeed, scholars of professional knowledge have long observed that, *“when it comes to practical knowledge acquired through experience, people cannot easily tell you what it is that they know”* (Eruat, 1994, p.25). Rather than relying on a method which asks people to articulate what they are doing, participants are given the vocabulary by which to express themselves; fixing this aspect of variance across participants offers increased methodological rigour and enhances replicability.

This study sought to answer the following questions:

(1) How do clinicians understand and regard the application of attachment theory and research within their routine clinical work in UK child mental health services?

(2) Where do clinicians’ understandings of attachment concepts align or misalign with those of researchers?

## Method

### Q methodology

Q methodology is a research method specifically designed to gain access to the richness, complexity and variety of human subjectivity and to subtle differences within and amongst different understandings on a given topic (Watts & Stenner, 2012). The approach requires participants to “sort” a set of items in an order of specified salience (e.g. agree/disagree or most likely/least likely). Each item is a statement relating to the topic of interest, and the complete set of statements is designed to represent the “concourse” of debate and observation around the topic. In producing a Q sort each participant provides a snap-shot of their overall viewpoint on the topic. The advantage is that each viewpoint/Q sort has a numerical expression that can be directly compared with those of the other participants (in the form of a correlation between complete Q sorts). The “by-person” correlation matrix is then analysed to yield a smaller number of factors, each of which identifies several significantly intercorrelating Q sorts/viewpoints. This is called a *by-person* factor analysis because the data for correlation and factoring are the complete Q sorts of individual participants (conventional factor analysis, by contrast, deals with correlations between items, not persons). This technical difference (which means that in Q participants are the *variables* and items are the *cases*) informs a broader theoretical difference: in Q the items do not measure the participant subjected to them, rather the participant lends meaning to the items from their perspective. This feature makes Q suitable for accessing and assessing a variety of opinions, beliefs, and perspectives. Q seeks to identify the range of viewpoints (*not* the spread of views across a population) particularly on topics over which there is debate. It allows shared viewpoints to be identified from the “bottom up” (i.e. a factor emerges *if* some of the provided Q sorts correlate together sufficiently enough to “load” that factor), but it also allows identification of differences between perspectives (i.e. assuming more than one factor emerges). Agreement in the context of Q does not establish that observations are accurate in an absolute sense, but rather that sorters have performed similarly in their organisation of the provided information/statements.

The aim of Q methodology is therefore to capture, describe, and explain patterns that are expressed within a small and strategically sampled cohort of participants, which permit certain generalisations of “concepts, categories, theoretical propositions and models of practice” (Watts & Stenner, 2012, p.89). Sampling is typically achieved theoretically, purposively or via non-random self-selection, according to criteria of interest, and – by virtue of the inverted approach – does not require large participant sample sizes in order to obtain insightful findings. Each participant is recruited because their viewpoint matters in relation to the subject at hand (Watts & Stenner, 2012): it is in the researchers’ interest for the final participant group to avoid being *“unduly homogeneous”* (p.71). Participation selection therefore seeks to balance relevance, sufficient variability, and limited levels of bias.

For our purposes Q methodology is relevant on several grounds. Firstly, it is the researcher who is tasked with reviewing the discourses and condensing these ideas into a set of individual items about the subject matter. This is a particular advantage given the complexity of attachment discourse (see Duschinsky et al., 2021). Waters and Deane (1985) claim that through the Q-sorting process participants are *“forced to clarify distinctions and ambiguities that are more easily glossed over in designing rating scales”* (p.52), enabling a close inspection of understanding and nuance that may not otherwise be available.

Secondly, when participants load onto one factor and not another, this suggests perspectives that are divergent; this may be interpreted through the factor array first, and with reference to participant characteristics or associated variables of interest second. The analysis of individual differences in these variables may identify other shared factors between participants and elucidate potential reasons for both shared and different understandings of applying attachment theory and research. An interpretation of why certain individuals may or may not share viewpoints with others can then be offered.

Themes for the concourse were generated through a review of relevant literature, a workshop with applied professionals interested in attachment and discussions with the study steering group. Field notes made during training in the Adult Attachment Interview (Berkeley 2017) and the Strange Situation Procedure (London 2015) were also reviewed. Items were refined, seeking to retain those that were most relevant, appropriately constructed, and offered adequate coverage of the topic area. The final set was piloted at an early stage with two renowned attachment theorists and two clinicians who were not part of the final sample. In the end, 65 items were retained for the final Q-set.

## Participant sample

This study was granted ethical approval from the Psychology Research Ethics Committee at the University of Cambridge. Thirty child health clinicians (six male, 24 female) in the UK, and 31 international experts in attachment research (10 male, 21 female) participated in the study. The appropriate number of participants needed for a Q-sort task is widely discussed, however it is generally agreed that the number of participants recruited should be smaller than the number of items in the concourse and typically between 20 and 60 participants (McKeown & Thomas, 2013; Stainton Rogers et al., 1995).

*Clinicians*. The study was advertised to clinical staff within two mental health Trusts in the UK with the following inclusion criteria: participants must be a registered health professional with at least one year’s clinical experience since qualification and be currently working as a psychologist or delivering psychological interventions. Clinical experience of the recruited practitioners spanned the range of 1–30+ years of professional practice. Clinical professionals worked across primary care (n = 7, 23.3%), secondary care (n = 11, 36.6%), and specialist services (n = 12, 40%). Ten clinicians (33.3%) were qualified Clinical Psychologists, 12 (40%) were Accredited Therapists (7 Systemic therapists, 1 Art therapist, 1 Cognitive-Behavioural Therapist, 1 Counselling Therapist, 1 Play Therapist, and 1 Integrative Therapist) and the remaining eight (26.6%) were other Allied Health Professionals (3 Nurses Practitioners, 3 Social Workers, 1 Wellbeing Practitioner, and 1 Occupational Therapist). Participating clinicians met with the researcher in person to complete the card sorting task so that accurate understanding and completion of the task could be supported.

*Researchers*. Researchers were purposively recruited for their expertise in attachment theory and research and/or the application of attachment theory and research in clinical practice. As active researchers within the field of attachment, the project steering group helped to compile a list of researchers to invite to participate in the study. A total of 77 invitations were sent out to potential participants. Since many were based abroad and mostly already familiar with Q-methodology, all researchers completed the study online using the platform Q-assessor (https://q-assessor.com). Of those who took part, 26 of those were developmentalists (50% take-up) and 5 were social psychologists (20% take-up). The majority of recruited researchers had experience of working in clinical practice (n = 17, 54.8%) and/or were involved in training clinicians (n = 21, 67.7%). A large majority (n = 25, 80%) were trained in either of the Strange Situation Procedure (Ainsworth, 1978), the Adult Attachment Interview (George, Kaplan and Main, 1985), or both, and a further nine (29%) were trained to deliver an evidence-based attachment-specific clinical interventions.

## Procedure

**Figure 1.**
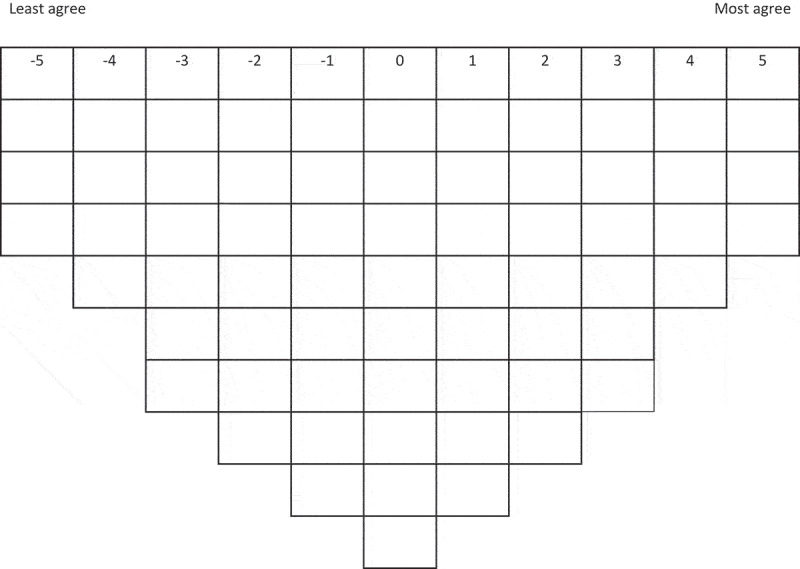
Q-sort distribution grid.

Participants were provided with the Q-set of 65 items and asked to initially sort the items into three piles: “largely agree,” “largely disagree,” and “not sure/no strong opinion.” They then assigned weightings to each item to sort them into a fixed, quasi-normal distribution grid (see Figure 1). A fixed distribution is typically used as it offers the most pragmatic means of facilitating the item ranking process (Watts & Stenner, 2012). Participants were informed that the specific order of items within columns was unimportant. All sorting decisions were preliminary and could be changed: sorts were finalised only when participants declared/decided they were finished.

## Results

Sixty-one completed sorts were entered into the statistical program *PQ Method* and subject to centroid factor analysis. A three-factor solution rotated by the varimax criterion best fit the data, with each factor demonstrating high reliability and low standard error. Many participants loaded onto more than one factor (known as co-loading and thus producing a highly correlated solution). The same was true of alternative solutions indicating that factors were not wholly distinct from each other and suggests the analysis offers a description of nuances in perspectives held within the population sampled. Nonetheless, there were clear differences across the defining sorts of each of the three factors. In addition, most of the distinguishing items were highly significant p < 0.01, indicating strong discrepancies between the factors on a number of specific items. The three-factor solution explained 55% of the total variance (Table 1).

**Table 1.** Factor solution characteristics.

	Factor 1	Factor 2	Factor 3
Defining sorts	13	17	17
Average reliability coefficient	0.80	0.80	0.80
Composite reliability	0.98	0.99	0.99
Eigenvalue	27.80	4.18	1.94
S.E. of factors	0.14	0.12	0.12
% Explained variance	17	19	19
Correlations	-	0.74	0.78
	0.74	-	0.62
	0.78	0.62	-

NB. Low-loading sorts pertained to eight researchers and six clinicians.

### Convergence

In this study, all sorts had at least one statistically significant factor loading (>.32), and 57% sorts (n = 35) had two or more statistically significant factor loadings. Defining sorts were selected as those where the highest loading squared accounted for more than 50% of the sum of the three loadings (Appendix 2 shows the final factor matrix loadings for the full dataset, including defining sorts in bold). Using these standard criteria, 77% of sorts (n = 47) were considered to be defining sorts, i.e. sorts that were included in the calculation of factor estimations. There were 24 significantly distinguishing items for Factor 1, 30 for Factor 2, and 23 for Factor 3 (see Appendix 1). Yet the majority of defining sorts were confounded: 31 sorts (51% of total) had multiple significant loadings compared with 16 (26% of total) defining sorts that had uniquely significant loadings. This pattern was largely similar across the subgroups and suggests a high degree of consensus. This supports the impression that the different factors represent differences in detail rather than fundamental disagreements in perspective, with a large body of shared understanding. Table 2 details the identified consensus statements, i.e. items that did not significantly distinguish between any pair of factors.

**Table 2.** Consensus statements: item scores and z-scores (rank order).

Item	F1 Sc	F2 Sc	F3 Sc	F1 z-score	F2 z-score	F3 z-score
34. Attachment patterns represent a child’s best attempt to deal with the caregiving environment, whether good or bad	+5	+5	+5	1.89	1.9	1.6
52. Attachment is a property of a relationship rather than a child	+4	+5	+5	1.74	2.09	1.78
48. Attachment theory is an important framework for making decisions about fostering placements and adoption	+4	+3	+4	1.49	1.26	1.32
61. Attachment concepts can be used to facilitate personalised care for children	+3	+4	+4	1.26	1.47	1.49
12. Attachment theory could be used more precisely within mental health practice	+3	+3	+3	1.03	1.09	1.2
47. Clinical interventions should be adapted to suit different attachment patterns	+3	+2	+3	1.28	0.91	1.14
32. Children’s attachment patterns can be predicted from knowledge of their parent’s attachment patterns	+1	+2	+1	0.44	0.75	0.76
3. The Coventry Grid is helpful for distinguishing attachment-related behaviours and autism-related behaviours	+1	+1	0	0.31	0.12	−0.05
55. Attachment disorders can be effectively treated with clinical intervention	+1	+2	+2	0.42	0.76	0.76
39. Attachment concepts are usually discussed when children don’t clearly fit a diagnostic category	0	0	0	0.03	−0.1	−0.18
25. Callous and unemotional traits in children originate from their early attachment experiences	0	−1	0	−0.1	−0.16	0.14
7. ADHD symptoms make it difficult to interpret attachment assessments	−1	−2	−1	−0.37	−0.61	−0.74
54. Attachment assessments focus specifically on where children direct their attention	−1	0	0	−0.24	−0.09	−0.09
57. Attachment assessment tools are easily accessible to clinicians	−2	−3	−2	−0.85	−0.95	−0.82
33. Children show the same attachment patterns across all their relationships	−3	−4	−4	−1.3	−1.56	−1.22
6. Attachment assessments cannot be used for children with autism spectrum disorder	−3	−3	−2	−0.97	−1.13	−0.79
26. Children with anxiety problems always have poor attachment relationships	−4	−2	−3	−1.37	−0.94	−1.00

Consensus items of particular interest are those with the same or similar item scores at the extreme ends of the rating scale. Despite often being the easiest items for individuals to sort, agreement with others about which items are placed at the extremes is harder to achieve. For instance, Table 2 shows items 34 and 52 are strongly endorsed by all the various clinicians and researchers sampled [+4, +5]. This highlights widespread agreement on the adaptive (attachment patterns represent a child’s best attempt to deal with the caregiving environment, whether good or bad) and dyadic (attachment is a property of a relationship rather than a child) elements of the attachment concept and can be regarded as two of the most important features of attachment theory for this context of applied practice. Similarly, there is strong consensus with respect to the perceived value of attachment theory as a framework for decision-making around fostering and adoption placements (48) and personalised care (61), offering specificity to domains of practice considered to be of particular relevance. In addition, there is consensus regarding what the attachment concept is *not* [−2, −3, −4], i.e. not a trait characteristic across all of a child’s relationships (33), not exclusive to neuro-typical children (6), and not always related to childhood anxiety (26). Other consensus items also offer nuances on the shared perspective held by participants. For instance, there is moderate agreement [+2, +3] amongst clinicians and researchers alike that attachment assessment tools are not easily accessible to clinicians (57), that attachment theory could be used more precisely within mental health (12), and that clinical interventions should be adapted to suit different attachment patterns (47).

**Table 3.** Distinguishing items for Factor 2.

Item	F2 rating	F2 z-score
9. Parent-child separations in a clinical setting can be used as part of an assessment	+5	1.48*
38. Good quality care throughout childhood is a better predictor of future mental health than a child’s early attachment pattern	+4	1.43*
45. The most effective attachment interventions target maternal sensitivity	+4	1.39*
2. Only validated assessments of attachment should be used in practice	+3	1.14*
8. The adult attachment interview is helpful to use with parents of children in services	+3	0.92
46. Interventions should target parents’ internal working models of relationships because this benefits their children	+2	0.89
16. Attachment concepts are useful to provide a sense of family dynamics	+2	0.85*
19. There is a high level of agreement amongst researchers about what attachment is	+2	0.58*
20. Childhood attachment patterns should not be used to predict adolescent outcomes	+1	0.50*
5. Attachment language is more helpful for clinical practice than specific attachment measures	+1	0.31*
42. Attachment theory is helpful for differential diagnosis in child mental health	+1	0.18*
41. Attachment disorders are over-diagnosed at the expense of other disorders	+1	0.19*
17. My colleagues understand what attachment is	+1	0.13*
51. Early attachment experiences determine how the brain develops	0	0.12*
24. Childhood attachment patterns are good predictors of adult relationship functioning	0	−0.11*
23. The disorganised attachment classification is the most relevant for mental health	0	0.06
30. Children’s mental health problems are often attachment-related problems	0	0.03*
36. Addressing attachment insecurity is a clinical priority because it leads to mental health problems later in life	−1	−0.24*
15. Too much focus is placed on attachment theory compared to other theories of child development	−1	−0.19*
10. The best way to work with attachment problems is to try a short intervention and see what happens	−1	−0.18*
40. Attachment problems and attention problems are often related	−1	−0.13*
21. It is impossible to develop a secure attachment to maltreating caregiver	−2	−0.88*
44. Knowing a child’s attachment status determines the type of treatment they need	−2	−0.76*
14. Insecure attachment is a research concept with little-to-no clinical application	−2	−0.46*
28. Attachment disorders are common in children	−3	−1.26*
1. Disorganised attachment can be used to identify child maltreatment	−4	−1.61*
53. The temperament of a child heavily influences the type of attachment they form	−4	−1.49*
4. Attachment is only relevant for children in fostering and adoption services	−4	−1.40*
37. After the first 1000 days attachment patterns are fixed for life	−5	−2.06
62. Childhood trauma lies behind all cases of attachment disorganisation	−5	−1.62*
63. Children with insecure attachments don’t want to be close to their attachment figures when they are ill or frightened	−5	−1.92*

NB. All items are significantly distinguishing at *p* < .05 level; * *p* < .01.

### Divergence

Factors 1 and 3 were considered to reflect the range of clinicians’ perspectives, as all clinician sorts loaded significantly onto at least one of these factors (>0.32). Factor 2 can be characterised as representing the defining factor for attachment researchers, as all but two (29/31) researcher sorts loaded significantly here (>0.32). The two researcher sorts that did not significantly load on factor 2 were both from participants that actively conduct applied research on clinical practice, unlike most of the other researchers. Here, Factor 2, endorsed uniquely by researchers, is presented first, followed by data discussing the divergence amongst clinicians. Appendix 3 presents interpretive summaries of the factors.

#### Factor 2

Seventeen participant sorts defined Factor 2, accounting for 19% of the variance in this solution. Table 3 shows the statistically distinguishing features of Factor 2.

**Table 4.** Distinguishing items for Factor 1.

Item	F1 rating	F1 z-score
50. Attachment patterns provide information about the function of behaviour	+5	1.81
17. My colleagues understand what attachment is	+3	1.13*
5. Attachment language is more helpful for clinical practice than specific attachment measures	+3	1.25*
36. Addressing attachment insecurity is a clinical priority because it leads to mental health problems later in life	+2	0.57
38. Good quality care throughout childhood is a better predictor of future mental health than a child’s early attachment pattern	+2	0.56
65. Working on a child’s attachment cannot be done without the involvement of their caregivers	+1	0.34*
46. Interventions should target parents’ internal working models of relationships because this benefits their children	+1	0.23*
8. The adult attachment interview is helpful to use with parents of children in services	0	0.21*
53. The temperament of a child heavily influences the type of attachment they form	0	−0.01*
20. Childhood attachment patterns should not be used to predict adolescent outcomes	0	−0.02*
23. The disorganised attachment classification is the most relevant for mental health	−1	−0.35
44. Knowing a child’s attachment status determines the type of treatment they need	−1	−0.17*
19. There is a high level of agreement amongst researchers about what attachment is	−1	−0.12*
15. Too much focus is placed on attachment theory compared to other theories of child development	−2	−0.95
13. Bowlby’s ideas are outdated for current clinical practice	−2	−0.88
10. The best way to work with attachment problems is to try a short intervention and see what happens	−2	−0.79*
62. Childhood trauma lies behind all cases of attachment disorganisation	−2	−0.65*
35. An attachment disorder diagnosis ensures children access the specialised attachment interventions that they need	−3	−1.2
43. A diagnosis of attachment disorder means the child has been unable form any kind of attachment relationship to a specific person/caregiver	−3	−1.18
29. It is rare that children develop secure attachments to parents with learning disabilities	−4	−1.53*
26. Children with anxiety problems always have poor attachment relationships	−4	−1.37
63. Children with insecure attachments don’t want to be close to their attachment figures when they are ill or frightened	−4	−1.35*
22. Children with severe learning disabilities cannot develop secure attachments	−5	−1.86*
37. After the first 1000 days attachment patterns are fixed for life	−5	−1.65

NB. All items are significantly distinguishing at *p* < .05 level; * *p* < .01.

Factor 2, as defined by attachment researchers, was characterised by a strong sense of what is important for childhood attachment: consistency of good quality care throughout childhood (38, +4) and high levels of maternal sensitivity (45, +4). In addition, a clear idea about how to assess for attachment in clinical practice, i.e. using robust validated attachment measures (2, +3), which should include parent-child separations (9, +5) and the Adult Attachment Interview (8, +3). Researchers did not consider attachment disorders to be common in children (28, −3), nor did they consider the development of a secure attachment to a maltreating caregiver to be an impossible task (21, −2). They considered attachment classifications to be necessary but perhaps insufficient (14, −2) for identifying suitable treatment plans (44, −2), characterised by their endorsement of the vaguer notion that attachment concepts are useful for gaining a sense of family dynamics (16, +2).

Researchers expressed solid endorsement of the direct use of specific attachment assessment measures in practice environments. However, they were also distinguished by their hesitancy to express strong claims regarding the utility of attachment theory for other clinical tasks/dilemmas such as: differential diagnosis (42, +1); diagnostic overshadowing (41, +1); prioritising interventions for attachment insecurity in an attempt to prevent later pathology (36, −1); identifying co-morbid problems of attention (40, −1); and the relative importance of attachment theory for clinical work compared with other theories of child development (15, −1). Researchers were also hesitant to endorse the ability to predict adolescent outcomes (20, 1) or adult relationship functioning (24, 0) from childhood attachment patterns in the context of clinical practice. In addition, they strongly rejected the idea that attachment patterns are largely fixed (37, −5) and definitive of later life relationships. They also disagreed with the position that temperament likely played an influential role in the development of childhood attachment (53, −4).

Finally, researchers were distinguished from clinicians by their strong opinions on the role and nature of the attachment disorganisation classification. For instance, they strongly disagreed with the notion that disorganised attachment can be used to identify child maltreatment (1, −4). This item produced two of the four largest discrepancies in factor scores (difference >±2 in item z-scores) across the whole analysis and was a highly distinguishing aspect of the *academic* perspective. Relatedly, there was a sharp disagreement with the notion that childhood trauma lies behind all cases of attachment disorganisation (62, −5). In fact, responses to this item were relevant to distinguishing all three factors, indicating moderate disagreement in Factor 1 (−2) in contrast to moderate agreement in Factor 3 (+2).

Overall, factor 2 presented a stance in line with key empirical, and especially meta-analytic, findings and strong views on a number of recent controversies in the literature (e.g. neurobiological underpinnings, attachment disorganisation, and attachment in the context of child maltreatment).

#### Factor 1

Twelve Clinicians and one researcher-clinician defined Factor 1, accounting for 17% of the variance in this solution. Table 4 shows the significantly distinguishing characteristics of Factor 1.

This factor was characterised by strong endorsement [+5] of attachment patterns offering information about the function of childhood behaviour (50), and a strong rejection of the idea that attachment theory was an inapplicable framework for parents (29) and children (22) with learning disabilities [−5 and −4, respectively]. They shared some similarities with the researcher factor, including strong rejections [−5] of attachment patterns as being fixed over time (37) and the idea that children with insecure attachment [−4] did not want to be close to their caregiver when ill or frightened (63). In contrast to researchers, they were less sure that good quality care is a better predictor of future mental health than early attachment patterns (38). The concept of parental sensitivity in caregiving behaviour has been widely used and supported as relevant despite Ainsworth’s scale never having been published (Duschinsky, 2020). Yet these findings suggest these clinicians consider sensitivity as less critical than researchers, perhaps due to different conceptual understandings or because it has been less marketed outside the field of attachment research. Clinicians here disagreed [−2] that too much focus was placed on attachment theory at the expense of other theories of child development (15), rather, suggesting that attachment is not considered enough within clinical settings. In particular, clinicians reflected that the attachment lexicon offered greater value [3] than formal attachment measures (5), not least because these measures are perceived as inaccessible.

However, participants in this factor were also characterised by uncertainty [−1, 0, +1] about whether to actually use some aspects of attachment theory and research for applied practice, such as: internal working models (46), the adult attachment interview (8), identified attachment patterns in treatment planning (44), the emphasis on dyadic interactions and dyads as a key target for intervention (65) and the predictive ability for broader adolescent outcomes. Items ranked in the middle of the distribution can be difficult to interpret. For instance, these may reflect pragmatic constraints on their individual skillset, service provision, or client group. Alternatively, this may reflect a true sense of uncertainty that may be due to either i) an unawareness, ii) inability to recall or iii) lack of available empirical findings.

Overall, Factor 1 expressed a sense that there were indeed valuable and worthwhile aspects to draw on in attachment theory and research but practitioners remained characteristically unsure about the use of some concepts and measures, often due to the realistic constraints of doing so. Demographic characteristics pertaining to these sorts revealed the majority of Clinical Psychologists sampled endorsed this viewpoint. Participants endorsing this point of view referenced first learning about attachment theory through developmental psychology. For these reasons it might be termed a *pragmatic and developmental perspective* on applications of attachment theory and research.

#### Factor 3

Seventeen participants defined Factor 3, accounting for 19% of the variance in this solution. Table 5 shows the significantly distinguishing characteristics of Factor 3.

**Table 5.** Distinguishing items for Factor 3.

Item	F3 rating	F3 z-score
8. The adult attachment interview is helpful to use with parents of children in services	+4	1.33
46. Interventions should target parents’ internal working models of relationships because this benefits their children	+3	1.23
64. Working in an attachment-informed way means providing a safe environment for children to explore	+3	1
36. Addressing attachment insecurity is a clinical priority because it leads to mental health problems later in life	+2	0.95
58. Attachment interventions work as well for adolescents as they do for younger children	+2	0.81*
62. Childhood trauma lies behind all cases of attachment disorganisation	+2	0.80*
31. Experiencing parental abuse or neglect will inevitably disrupt a child’s ability to form a secure attachment	+2	0.78*
44. Knowing a child’s attachment status determines the type of treatment they need	1	0.30*
60. All children in mental health services who lack secure attachments need attachment-informed interventions	1	0.63*
38. Good quality care throughout childhood is a better predictor of future mental health than a child’s early attachment pattern	0	0.2
56. Attachment assessments enable practitioners to clearly separate innate factors from environmental factors	0	0.10*
11. Attachment classifications over-simplify differences between people	0	−0.50*
19. There is a high level of agreement amongst researchers about what attachment is	−1	−0.62*
17. My colleagues understand what attachment is	−1	−0.53*
18. Attachment research literature is difficult to translate into clinical practice	−1	−0.76*
63. Children with insecure attachments don’t want to be close to their attachment figures when they are ill or frightened	−1	−0.66*
53. The temperament of a child heavily influences the type of attachment they form	−1	−0.65*
59. There is so much literature on attachment it is difficult to know which bits are most useful for practice	−1	−0.65*
5. Attachment language is more helpful for clinical practice than specific attachment measures	−2	−0.91*
20. Childhood attachment patterns should not be used to predict adolescent outcomes	−2	−0.82*
23. The disorganised attachment classification is the most relevant for mental health	−3	−0.97*
37. After the first 1000 days attachment patterns are fixed for life	−3	−1.2
10. The best way to work with attachment problems is to try a short intervention and see what happens	−4	−1.42*
15. Too much focus is placed on attachment theory compared to other theories of child development	−4	−1.35
27. Attachment insecurity in children is so common it is not problematic by itself	−5	−1.44*

NB. All items are significantly distinguishing at *p* < .05 level; * *p* < .01.

Factor 3 was characterised by a strong sense [−5] that attachment insecurity is a key cause for concern in its own right (27) and they did not believe [−3] the disorganised attachment classification was the most relevant for mental health (23). Interestingly, throughout the sorting process many of these clinicians spoke of their aversion to pathologizing language in general in the context of attachment, disparaging terminology of attachment “disorder” or “disorganisation.” These participants felt strongly [−4] that not enough attention in child services is given to attachment theory (15) and were unsure how to evaluate whether it offers an oversimplified framework (11). Participants also expressed a high valuation of the use of formal attachment assessment measures in their work (5).

The Factor 3 perspective expressed uncertainty [−1] surrounding whether a child with an insecure attachment wished to be close to their caregiver when ill or frightened (63). From a theoretical point of view this appears to downplay the behavioural-systems understanding of attachment, in favour of a perception of insecurity as disturbing even the wish to be close. With respect to interventions for attachment insecurity, participants were hesitant [+1] to claim that an attachment-informed intervention was always required (60) but felt strongly [−4] that short interventions with these cases were inappropriate (10).

Within this point of view, participants also felt very strongly [−5] that Bowlby’s ideas were not outdated for clinical practice (13), and they were characteristically unsure [0] about the good quality care being a better indicator of future mental health than early attachment patterns (38). In summary, this suggests a key feature of this perspective is concern pertaining to the adverse nature of early insecurity.

Overall, Factor 3 conveyed an understanding of attachment through strong expression of the detrimental nature of attachment insecurity and the ongoing relevance of addressing this in a therapeutic way. Participants emphasised the role of mental representations, internal working models, and past experiences of attachment, seeing great value in the Adult Attachment Interview. In contrast to the other two factors, there was less expression of a behavioural systems-level understanding of attachment and a notably different perspective on matters of attachment disorganisation and childhood trauma. A two-tailed independent t-test found that clinicians loading onto Factor 3 had significantly more clinical experience working with children, adolescents, and their families (M = 16.58 years), compared to those loading on Factor 1 (M = 7.45 years), t(17) = 2.49, p = .02. Participants endorsing Factor 3 were frequently systemic psychotherapists, but also a few clinician-researchers. The majority attributed their learning of attachment to personal reading of clinically focused literature (e.g. Crittenden, 2016; Hughes, 2007; Dallos & Vetere, 2009; Golding et al., 2012), continuing professional development, and post-qualification training in specific modalities, rather than core professional training or traditional texts (e.g. The Handbook of Attachment, Cassidy & Shaver, 2016). For these reasons it was labelled an *autodidactic and therapeutic perspective*.

#### Key differences between perspectives

Table 6 shows the largest differences in z-scores between Factor 2 and the other two factors.

**Table 6.** Key differences between Factor 2 and both other factors (descending by z-score difference).

	Item scores		Factor z-scores		
Item	F2	F1	F2	F1	±Diff.
1. Disorganised attachment can be used to identify child maltreatment	−4	+1	−1.61	0.46	2.07
51. Early attachment experiences determine how the brain develops	0	+5	0.12	1.88	1.76
53. The temperament of a child heavily influences the type of attachment they form	−4	0	−1.49	−0.01	1.48
2. Only validated assessments of attachment should be used in practice	+3	−1	1.14	−0.13	1.28
45. The most effective attachment interventions target maternal sensitivity	+4	0	1.39	0.14	1.25
	F2	F3	F2	F3	±Diff.
62. Childhood trauma lies behind all cases of attachment disorganisation	−5	+2	−1.62	0.79	2.42
1. Disorganised attachment can be used to identify child maltreatment	−4	+1	−1.61	0.50	2.10
51. Early attachment experiences determine how the brain develops	0	+5	0.12	1.79	1.67
27. Attachment insecurity in children is so common it is not problematic by itself	0	−5	0.08	−1.44	1.52
20. Childhood attachment patterns should not be used to predict adolescent outcomes	+1	−2	0.50	−0.82	1.32

In line with, and perhaps reinforced by, the recent consensus statement led by Granqvist et al. (2017), researchers strongly disagreed with the notion that disorganised attachment can be used to identify child maltreatment (1, −4). This item produced two of the four largest discrepancies in factor scores (difference >±2 in item z-scores) across the whole analysis. Relatedly, another key characteristic of the academic perspective was a sharp disagreement with the notion that childhood trauma lies behind all cases of attachment disorganisation (62, −5). In fact, responses to this item were relevant to distinguishing all three factors, indicating moderate disagreement in Factor 1 (−2) and moderate agreement in Factor 3 (+2).

Of similar magnitude was the discrepancy found regarding the role of early attachment experiences in determining brain development (51): while both Factors 1 and 3 gave equally strong endorsement of this [+5], the researchers sorted this item firmly into the middle of the distribution [0], conveying a unique tentativeness about this claim. Finally, researchers strongly disagreed [−4] with the idea that child temperament heavily influences the type of attachment pattern formed, compared to Factor 1 [0] and Factor 3 [−1] who were unsure.

Table 7 shows the largest differences in z-scores between Factor 1 and Factor 3.

**Table 7.** Top ten differences between clinician perspectives (descending by z-score difference).

	Item scores		Factor z-scores		
Statement	F1	F3	F1	F3	±Diff.
5. Attachment language is more helpful for clinical practice than specific attachment measures	+3	−2	1.25	−0.91	2.17
17. My colleagues understand what attachment is	+3	−1	1.13	−0.53	1.66
62. Childhood trauma lies behind all cases of attachment disorganisation	−2	+2	−0.65	0.80	1.45
27. Attachment insecurity in children is so common it is not problematic by itself	0	−5	−0.10	−1.44	1.34
8. The adult attachment interview is helpful to use with parents of children in services	0	+4	0.21	1.33	1.12
11. Attachment classifications over-simplify differences between people	+2	0	0.60	−0.50	1.09
46. Interventions should target parents’ internal working models because this benefits their children	1	3	0.23	1.23	1.10
59. There is so much literature on attachment it is difficult to know which bits are most useful for practice	+1	−1	0.28	−0.65	0.93
20. Childhood attachment patterns should not be used to predict adolescent outcomes	0	−2	−0.02	−0.82	0.80
50. Attachment patterns provide information about the function of behaviour	+5	+3	1.81	1.07	0.74

Participants endorsing Factor 1 indicated a particular preference [+3] for using the lexicon of attachment theory and considered a behavioural level understanding attachment patterns (50) to be particularly helpful [+5], yet cautioned that the classification system (11) represented somewhat of an oversimplification [+2]. By contrast, participants aligned with Factor 3 anticipated greater utility of specific attachment measures (5), in particular a strong optimism [+4] about the use of the adult attachment interview for routine clinical practice (8), and perceived value [+3] of therapeutically targeting parents’ internal working models of attachment (46) that may indicate a preferential understanding of attachment at the level of cognitive representation. Finally, whereas Factor 1 participants did not necessarily consider attachment insecurity to be problematic, Factor 3 participants felt very strongly [−5] that it should not be ignored and expressed uncertainty about how well their colleagues really understood attachment concepts.

## Discussion

### Similarities and differences

The overarching aim of this study was to examine how clinicians working with children and attachment researchers understand attachment theory and research in the context of clinical practice, using Q methodology to identify areas of convergence and divergence, and interpret commonalities between those holding shared perspectives. There were significant commonalities between researchers and clinicians, perhaps more than would be expected based on previous impressions derived from document analysis (Duschinsky, 2020; White et al., 2020). Points of agreement typically favoured matters of service practicalities (e.g. accessibility and appropriateness of tools) and general theoretical tenets (e.g. attachment patterns are malleable and dyadic in nature). One of the sharpest points of agreement was the more sophisticated perception regarding the nature of attachment: participants perceive that attachment patterns are shaped in response to experiences of caregiving and are adaptive to these environments. Notably, clinicians are researchers are united in their vision for clinical interventions to be tailored to suit the needs of different attachment classifications and in their recognition that precision in this area is currently lacking.

However, there was also some key examples of divergent understanding, and this study makes an important contribution to the literature by being able to specify the issues of disagreement between clinicians and researchers. The most striking differences of opinion related to claims made about attachment disorganisation and trauma. The perception that trauma experiences underpin attachment disorganisation and the use of the latter as a proxy for the former were strongly rejected by the research community. By contrast, clinicians on both factors expressed a lack of strong opinion about identifying maltreatment in this way. This was the largest identified area of divergence. Yet the finding may be a consequence of an earlier part of the wider research programme, of which this study is a part, that led to the Granqvist et al. (2017) consensus statement on disorganised attachment. The result observed here may in fact reflect the ability of the research community to respond to such consensus-building attempts, as well as suggesting that there is still some way to go in sharing this updated understanding with practice communities.

Views about the implications of early attachment experiences on children’s brain development were also markedly different between researchers and clinicians. Clinicians strongly endorsed these claims whilst researchers were characterised by their wariness to draw conclusions in either direction. Perhaps, as some have speculated (e.g. White et al., 2020), emphasising associated neurobiology appears to offer scientific credibility to the observations and intuitions of practitioners working with, and needing to make decisions based upon, children’s attachment. Alternatively, neurobiology may appear to characterise something affecting the very “core” of individual experience and potentially point to a mechanistic explanation, relevant to practitioners. Both implications can be drawn from the work of Schore (2001), which has been popular with clinical audiences. Certainly researchers have made significant headway in exploring attachment and neuroscientific correlates. For example, Long et al. (2020) recently presented a neuro-anatomical model of human attachment from their summary of the available neuroscience data. Yet generally researchers acknowledge that, overall, science has “only just scratched the rather impenetrable surface of this elusive association” (van IJzendoorn et al., 2021, p.200). We consider that the appeal of neuroscientific research findings remains highly attractive to stakeholders outside of the academe and that transmission gaps of this nature also exist outside of attachment theory.

Finally, discrepancies regarding suitable treatment targets were identified. Researchers were strongly in favour of maternal sensitivity as the most effective target for attachment-based interventions, whereas clinicians did not endorse this. Based on analysis of published texts, Duschinsky (2020) has concluded that, outside developmental psychology, Ainsworth’s technical use of the concept of sensitivity is little recognised. The ordinary language connotations risk portraying sensitive caregiving as a fluffy “extra” rather than a crucially determining factor linking caregiving to child socioemotional development and mental health. Instead of sensitivity, clinicians aligning with the *autodidactic and therapeutic perspective* favoured targeting parents’ internal working models as a target of treatment for their children’s wellbeing. Duschinsky’s (2020) analysis states that this was a common position among attachment researchers in the 1990’s and has subsequently featured throughout the psychotherapy-orientated books directed to clinicians. Additionally, he highlights that meta-analyses have been typically excluded from the literature directed to psychotherapists, and thus the finding from Bakermans-Kranenburg et al. (2003) that interventions targeting internal working models were less effective than those targeting interactions is unlikely to be known by these clinicians but will have been salient to researchers.

### Clinical implications: is attachment a diagnostic issue?

The international community of attachment researchers have recently engaged in a debate related to the question of how a dyadic concept can fit with diagnosis-based healthcare, after Lyons-Ruth and Jacobvitz (2016) argued for the revalidation of Ainsworth’s classification system, particularly the disorganised classification, into the currency of diagnostic categories for ease of application to practice. However, others in the field have argued strongly against such a solution (see Zeanah & Lieberman, 2016) and, as yet these proposals have not been taken forward. This study found clear agreement that attachment patterns are to be understood to reflect a quality of a dyadic relationship, rather than an individual characteristic. However, dyadic constructs are somewhat foreign to the current set up and design of health services and it is a struggle to meaningfully integrate these into practice. For instance, with the rare exception of the DSM-5’s Munchausen syndrome by proxy (also known as factitious disorder imposed on another in ICD-11), there are no other diagnostic categories in physical or mental health that refer to symptoms or characteristics pertaining to more than one individual, and only a couple of other clinical descriptions. The system is therefore without the necessary structures in place to identify, manage, or treat dyadic presentations. The American Zero to Three/National Center for Clinical Infant Programs (1994) was designed specifically for the purpose of assessing relationships, mental health, and development of young children and offers a potential exception to this, but it does not have the standing or institutional support of other more commonly used diagnostic manuals. The lack of attachment descriptions as formal diagnostic labels potentially renders this type of knowledge less likely to be understood or retained by the psychiatric or healthcare systems, which are ultimately organised around individual categories. This issue of diagnostic recognition/classification is of vital importance precisely because of its resulting effect on practice behaviour and understanding. Therefore, the introduction of attachment patterns as a diagnostic issue potentially places the dyadic quality of attachment at risk of conceptual and behavioural neglect or at least as a nodal point for divergent interpretations of the theory.

### Theoretical implications: are there different theoretical understandings in circulation?

During the sorting process, we identified that some clinicians attempt to avoid implying that attachment problems are located within children themselves by using less- or non-pathologising language in their communication of these concerns. Participants endorsing the *autodidactic and therapeutic perspective* strongly rejected what they regarded as the value-laden ideas of “attachment disorder” and “disorganised” attachment. This perspective was also characterised by a particularly heightened concern about attachment insecurity, so much so that the desire of children with insecure attachments to still want to be close to caregivers when ill or frightened was significantly underestimated compared to the other two perspectives. This point of divergence indicates that some clinicians lack the behavioural-systems level understanding of the attachment system that was expressed by the majority of participants in this study.

Whereas participants endorsing the *pragmatic, developmental and uncertain perspective* were almost exclusively Clinical Psychologists, it was mostly systemic and other psychotherapists who endorsed the *autodidactic and therapeutic perspective*. Notably, the latter factor consisted of participants with significantly more clinical experience than the former: an additional 9 years on average. We consider that the specific literature and teaching on attachment theory that professionals are exposed to as part of their professional training programs differs and may influence their subsequent perspectives about its application for practice. Their training and the kinds of challenges faced in their work may also influence the relative appeal of works by other therapists writing about, and offering commercial training on, the psychotherapeutic implications of attachment. When asked what texts they were familiar with as a supplemental question, many endorsing the *autodidactic and therapeutic perspective* made reference to therapeutically-orientated sources of literature from authors such as Patricia Crittenden, David Howe, Dan Hughes, Rudi Dallos, Miriam Silver, Dan Siegel, and Kim Golding. This literature appears to be both appealing to and targeted at systemic and other psychotherapists, more than Clinical Psychologists, and generally featured in post-qualification training courses rather than on undergraduate or postgraduate curriculums. A notable quality of these texts is that they typically portray attachment insecurity as the ultimate underlying mechanism of mental ill-health in general and so offer recommendations for working with problems associated with insecurity. This might explain the preponderance of expressed concern for attachment insecurity in the *autodidactic and therapeutic perspective*.

Both the clinical and theoretical implications of this work point to the issue of inaccessible attachment assessment tools for clinicians. One attempt to address this has been a collaborative effort from researchers and clinicians in Canada, to develop and validate an observational screening instrument for disrupted caregiving in community settings (AMBIANCE-brief, Cooke et al., 2020; but see Van IJzendoorn et al. (2018) discussing the risk of false positives and false negatives in translating group-level measures to individual screening or diagnoses). A possible benefit of this measure is that it may permit clinicians to bring into their assessment practice the dyadic view of attachment that this study shows is shared between clinicians and researchers. Secondly, the value of ongoing attempts to clarify the evidence-based terminology and lexicon of attachment theory (see Society for Emotion and Attachment Studies (SEAS), 2021) cannot be overstated, given the critical role of language in shaping both conceptual understanding and practice behaviour as evidenced here.

### Strengths and limitations

This is the first study to empirically examine clinician and researcher understandings of attachment knowledge for applied clinical practice and was conducted without prior assumptions about who held the authority on recommendations for practice. Crucially, this was done by collectively analysing the perspectives of clinicians and researchers together as analysing one or other of these stakeholder groups would have resulted in participants imposing their own understanding onto carefully phrased propositions in ways that could not be detected. This study has also generated some hypotheses about the impact of specific training opportunities, reading materials and clinicians’ professional trajectories as mediating factors for the perspectives that they hold, which offer interesting avenues for future research.

Q-sort data ultimately offers a one-time snapshot of participants’ relationship with discourse. What this study is unable to account for is how these identified perspectives may play out in examples of case discussion, clinical reasoning, and practice behaviour; it may be, for example, that different points of view come to the forefront under different circumstances. However, practice behaviour is specific to a given case and therefore investigations of this would have to choose whether to examine clinicians working on different cases or to provide a standard case-vignette. Assessments of vignette-based reasoning are currently underway by colleagues. Actual observations of practice in future research will contribute valuable insights to the study of practitioners’ use of attachment theory and research.

As was anticipated, none of the clinician participants recruited to this study were familiar with Q-methodology, compared with most of the researchers who were. It was therefore helpful to conduct the clinician sorts in-person, so that accurate understanding and completion of the task could be supported, and where consistency of item interpretation could be monitored. Two clinicians and two researchers agreed to complete the study via both methods (online vs in-person) to allow for cross-checking between their sorts. The impact of using different data collection methods for participant-groups did not have any identifiable impact on the observed results.

Finally, whilst this is the first empirical study to examine the perspectives of attachment researchers, limitations stem from the purposive sampling used. The study was undertaken within a research group known to be studying attachment in clinical and social welfare practice, and with closer links with developmental than with social psychology colleagues. This may account for the differential take up (50% of invited developmentalists vs 20% of invited social psychologists) and the relatively small number of social psychologists who completed the study (n = 5). Similarly, a recent interview study conducted by Spies and Duschinsky (2021) invited 39 researchers across both traditions. Fifteen agreed to be interviewed, all of whom were developmentalists. For participants, the present study may have been identifiable as being connected to the research group’s wider project (e.g. Duschinsky 2020; Duschinsky & Foster 2021), making some items in the concourse especially salient for colleagues and perhaps leaving participation vulnerable to demand characteristics. Two items relating to attachment disorganisation may have been particularly impacted by this, regarding the role of childhood trauma as a causal factor and the use of the classification to identify maltreatment. It is possible that some degree of social desirability contributed to the degree of difference observed on the latter item, but, more likely, it illustrates that the community of attachment researchers had been influenced by work towards and the publication of Granqvist et al. (2017). In addition, the differential take-up of researchers to participate in the study infers a potentially striking demand characteristic that may have resulted from the perception of the research group within the field.

## Conclusions

Despite extensive literature proposing what clinicians should do with attachment theory and research there has been almost no empirical investigation of how clinicians or researchers understand these concepts and their application to clinical practice, and indeed whether they align or conflict. This study used Q-methodology to capture the perspectives held and found they were highly correlated, with significant shared understanding regarding the nature of the attachment relationship and their vision for its role in applied practice. However, there were some distinct axes of divergence, on matters such as attachment disorganisation, brain development, treatment targets, and linguistic preferences, which may be in part because these groups operate with different connotations about the meanings of the attachment classifications and their assessment. There may be an extent to which the absorptive quality of attachment concepts parallels the conditional strategy of the avoidant attachment classification: in the immediate term, it “keeps the show on the road” (i.e. it maintains a degree of proximity to the caregiver; or sustains attention to attachment principles) but in the long-term this absorptive quality hinders genuine and specific engagement and dialogue, and perhaps also obstructs delivery of the degree of personalised care that clinicians and researchers aspire to.

## Correction Statement

HB led the project as part of her doctoral submission at the University of Cambridge and wrote the manuscript under the primary supervision of RD, with additional oversight from MvI, MF and MW on matters of conceptualisation and analysis. PS provided expert consultation on Q-methodology. All authors edited the final version of the manuscript. Data is available on request to the first author.

This article has been corrected with minor changes. These changes do not impact the academic content of the article.

## Appendix 1: Full item-set with distinguishing items highlighted

**Table:**

Statement	F1	F2	F3
1. Disorganised attachment can be used to identify child maltreatment	+1	**−4***	+1
2. Only validated assessments of attachment should be used in practice	−1	**+3***	0
3. The Coventry Grid is helpful for distinguishing attachment-related behaviours and autism-related behaviours	+1	+1	0
4. Attachment is only relevant for children in fostering and adoption services	−5	**−4***	−5
5. Attachment language is more helpful for clinical practice than specific attachment measures	**+3***	**+1***	**−2***
6. Attachment assessments cannot be used for children with autism spectrum disorder	−3	−3	−2
7. ADHD symptoms make it difficult to interpret attachment assessments	−1	−2	−1
8. The adult attachment interview is helpful to use with parents of children in services	**0***	**+3**	**+4**
9. Parent-child separations in a clinical setting can be used as part of an assessment	+2	**+5***	+2
10. The best way to work with attachment problems is to try a short intervention and see what happens	**−2***	**−1***	**−4***
11. Attachment classifications over-simplify differences between people	+2	+2	**0***
12. Attachment theory could be used more precisely within mental health practice	+3	+3	+3
13. Bowlby’s ideas are outdated for current clinical practice	**−2**	−3	−5
14. Insecure attachment is a research concept with little-to-no clinical application	−4	**−2***	−4
15. Too much focus is placed on attachment theory compared to other theories of child development	**−2**	**−1***	**−4**
16. Attachment concepts are useful to provide a sense of family dynamics	+4	**+2***	+4
17. My colleagues understand what attachment is	**+3***	**+1***	**−1***
18. Attachment research literature is difficult to translate into clinical practice	0	+1	**−1***
19. There is a high level of agreement amongst researchers about what attachment is	**−1***	**+2***	**−1***
20. Childhood attachment patterns should not be used to predict adolescent outcomes	**0***	**+1***	**−2***
21. It is impossible to develop a secure attachment to maltreating caregiver	0	**−2***	0
22. Children with severe learning disabilities cannot develop secure attachments	**−5***	−3	−3
23. The disorganised attachment classification is the most relevant for mental health	**−1**	**0***	**−3***
24. Childhood attachment patterns are good predictors of adult relationship functioning	+2	**0***	+2
25. Callous and unemotional traits in children originate from their early attachment experiences	0	−1	0
26. Children with anxiety problems always have poor attachment relationships	**−4**	−2	−3
27. Attachment insecurity in children is so common it is not problematic by itself	0	0	**−5***
28. Attachment disorders are common in children	−2	**−3***	−2
29. It is rare that children develop secure attachments to parents with learning disabilities	**−4***	−2	−1
30. Children’s mental health problems are often attachment-related problems	+2	**0***	+1
31. Experiencing parental abuse or neglect will inevitably disrupt a child’s ability to form a secure attachment	−1	−1	**2***
32. Children’s attachment patterns can be predicted from knowledge of their parent’s attachment patterns	+1	+2	+1
33. Children show the same attachment patterns across all their relationships	−3	−4	−4
34. Attachment patterns represent a child’s best attempt to deal with the caregiving environment, whether good or bad	+5	+5	+5
35. An attachment disorder diagnosis ensures children access the specialised attachment interventions that they need	**−3**	−1	−2
36. Addressing attachment insecurity is a clinical priority because it leads to mental health problems later in life	**+2**	**−1***	**+2**
37. After the first 1000 days attachment patterns are fixed for life	**−5**	**−5**	**−3**
38. Good quality care throughout childhood is a better predictor of future mental health than a child’s early attachment pattern	**+2**	**+4***	**0**
39. Attachment concepts are usually discussed when children don’t clearly fit a diagnostic category	0	0	0
40. Attachment problems and attention problems are often related	+2	**−1***	+1
41. Attachment disorders are over-diagnosed at the expense of other disorders	−3	**+1***	−3
42. Attachment theory is helpful for differential diagnosis in child mental health	+3	**+1***	+1
43. A diagnosis of attachment disorder means the child has been unable form any kind of attachment relationship to a specific person/caregiver	-**3**	−2	−2
44. Knowing a child’s attachment status determines the type of treatment they need	**−1***	**−2***	**1***
45. The most effective attachment interventions target maternal sensitivity	0	**+4***	+1
46. Interventions should target parents’ internal working models of relationships because this benefits their children	**+1***	**+2**	**+3**
47. Clinical interventions should be adapted to suit different attachment patterns	+3	+2	+3
48. Attachment theory is an important framework for making decisions about fostering placements and adoption	+4	+3	+4
49. Attachment behaviour only means something when a child feels threatened or anxious	−2	−1	−3
50. Attachment patterns provide information about the function of behaviour	**+5**	+3	+3
51. Early attachment experiences determine how the brain develops	+5	**0***	+5
52. Attachment is a property of a relationship rather than a child	+4	+5	+5
53. The temperament of a child heavily influences the type of attachment they form	**0***	**−4***	**−1***
54. Attachment assessments focus specifically on where children direct their attention	−1	0	0
55. Attachment disorders can be effectively treated with clinical intervention	+1	+2	+2
56. Attachment assessments enable practitioners to clearly separate innate factors from environmental factors	−3	−3	**0***
57. Attachment assessment tools are easily accessible to clinicians	−2	−3	−2
58. Attachment interventions work as well for adolescents as they do for younger children	+1	0	**+2***
59. There is so much literature on attachment it is difficult to know which bits are most useful for practice	+1	+1	**−1***
60. All children in mental health services who lack secure attachments need attachment-informed interventions	−1	0	**1***
61. Attachment concepts can be used to facilitate personalised care for children	+3	+4	+4
62. Childhood trauma lies behind all cases of attachment disorganisation	**−2***	**−5***	**+2***
63. Children with insecure attachments don’t want to be close to their attachment figures when they are ill or frightened	**−4***	**−5***	**−1***
64. Working in an attachment-informed way means providing a safe environment for children to explore	**+4***	+3	**+3***
65. Working on a child’s attachment cannot be done without the involvement of their caregivers	+1	+4	+3

NB. Distinguishing statements p<0.05 indicated in bold; * distinguishing statements p<0.01

## Appendix 2: Final factor matrix

**Table:**

Sort	F1	F2	F3	Sort	F1	F2	F3
C1	0.43	0.30	0.44	R1	0.44	0.44	0.04
C2	0.50	0.16	**0.55**	R2	0.14	**0.53**	0.31
C3	**0.56**	0.36	0.38	R3	0.15	0.58	**0.65**
C4	**0.58**	0.36	0.22	R4	0.04	0.49	**0.63**
C5	**0.62**	0.33	0.45	R5	0.41	**0.66**	0.34
C6	0.48	0.27	0.41	R6	0.31	0.47	0.56
C7	**0.63**	0.48	0.21	R7	0.33	**0.61**	0.50
C8	**0.58**	0.20	0.41	R8	0.41	**0.64**	0.35
C10	0.30	0.27	**0.47**	R9	0.19	**0.70**	0.16
C11	0.28	0.16	**0.53**	R10	0.25	**0.78**	0.06
C12	0.34	0.40	**0.56**	R11	0.26	0.52	0.47
C13	0.22	0.28	**0.66**	R12	0.37	**0.58**	0.34
C15	0.29	0.14	**0.58**	R13	0.12	**0.53**	0.43
C16	0.56	0.28	0.55	R14	0.16	0.50	0.51
C17	0.46	0.37	0.50	R15	0.38	0.41	0.15
C18	−0.02	0.20	**−0.45**	R16	−0.13	**0.63**	−0.22
C19	**0.58**	0.15	0.52	R17	**0.57**	0.41	0.21
C20	**0.60**	0.10	0.35	R18	0.46	0.50	0.22
C21	0.44	0.23	0.39	R19	0.32	**0.72**	−0.16
C22	0.50	0.39	0.31	R20	0.43	0.07	**0.59**
C23	**0.55**	0.43	0.12	R21	0.13	**0.76**	0.27
C24	**0.54**	0.10	0.50	R22	0.47	0.45	0.32
C25	0.43	0.11	**0.65**	R23	0.46	**0.52**	0.23
C26	**0.41**	0.16	0.33	R24	0.46	**0.72**	0.20
C27	0.31	0.07	**0.68**	R25	0.26	0.31	**0.72**
C28	0.26	0.07	**0.67**	R26	0.13	0.50	**0.61**
C29	**0.62**	0.43	0.38	R27	0.56	0.37	0.43
C30	0.19	0.18	**0.72**	R28	0.43	**0.61**	0.09
C31	**0.65**	0.15	0.41	R29	0.35	**0.54**	0.32
C32	0.31	0.03	**0.62**	R30	0.40	**0.64**	0.22
-	-	-	-	R31	0.42	**0.52**	0.17

NB. Defining sorts highlighted bold, non-defining sorts shaded grey. Significant factor loading ≥0.32, *p* < 0.01 Sorts labelled C# refer to clinician sorts and sorts labelled R# refer to researcher sorts.

## Appendix 3: Interpretive summaries

### Consensus statement

Attachment patterns represent a child’s best attempt to adapt to their environment (34) – they describe something important about the relationship an infant has with their caregiver (52). This is a really important framework for thinking about looked after children and in making decisions about fostering placements and adoption (48). Ideally, we should adapt clinical interventions to suit the individual needs of children with different attachment patterns (47) and offer personalised care (61). At the moment, this level of precision is not used enough (12). One reason for this is that attachment assessment tools are not easily accessible to clinicians (57). It is also complicated by the knowledge that children show different attachment patterns with different caregivers (33). But, if made possible, attachment assessments could be used with children across the neurodevelopmental spectrum (6).

There are some elements of attachment theory that we don’t yet have a good understanding of. For example, it’s not clear whether attachment assessments focus on where children direct their attention (54) and what implications this has for interpreting co-occurring symptoms of ADHD (7). We are not sure how well children’s attachment patterns can be predicted from knowledge of their parent’s attachment patterns (32). We are also unsure how well attachment disorders can be treated with clinical interventions (55) or whether this framework is more helpful for non-diagnostic considerations (39). Finally, we don’t know whether callous and unemotional traits in children originate from their early attachment experiences (25).

### Pragmatic, developmental, and uncertain perspective

Attachment theory was taught as part of my core professional training, primarily via the early work of John Bowlby and Mary Ainsworth. Done well, thinking about this carefully can help us tailor our approach to the individual needs of children (61, +3), but we probably do this in a slightly vague way (12, +3). It is tricky because children can have different sorts of attachment to different caregivers (33). Although I know attachment is about the relationship between a parent and child (52, +4), we don’t always get the opportunity to work with the relevant caregivers (65, +1) so we just do our best and talk about attachment generally. I understand that children with insecure attachments still want to be close to their caregivers when they are ill or frightened (63) they just show this differently to securely attached children.

My colleagues understand what attachment is about (17, +3) and being able to talk about child behaviour and family dynamics (16, +4) with them in this way is helpful (5, +3). People used to worry about whether children (22) and/or parents (29) with learning disabilities could still develop secure attachments, but in my experience they absolutely can. Parents tell us some children seem to be born temperamentally different, and maybe that affects attachment but I’m not sure how (53, 0). But attachment patterns are definitely not fixed for life (37, −5) and good quality care throughout childhood is probably more important overall (38, +2). I am certain, though that early attachment experiences shape how the brain develops (51, +5) and this is partly why Bowlby’s ideas are really relevant (13).

One of the most useful things about attachment is that it helps us to understand child behaviour (50, +5), but other concepts are less obviously applicable. For example, I don’t how relevant parents’ internal working models are to their children’s attachment (46, +1) and without understanding this or having access to specific assessments tools (57, −2) I couldn’t say whether the adult attachment interview is useful or not (8, 0). Sometimes we can work out a child’s attachment classification (even without the validated measures), but this doesn’t necessarily help to identify what intervention they need (44, −1), and, unfortunately, even an attachment disorder diagnosis doesn’t necessarily ensure that a child gets access to specialised interventions (35, −3). Not every child, for example, with anxiety problems also has poor attachment relationships (26, − 4), so we consider attachment alongside other theories of child development (15) and mental health. Sometimes addressing attachment insecurity looks like the clinical priority, (36, +2) but we are not really commissioned to do that kind of work in our services so we are limited in how much we can really focus on it routinely.

### Academic perspective

The most important thing for child development is good quality care throughout childhood (38, +4). This means that interventions must focus on improving parental sensitivity as the active ingredient (45, +4). Bowlby proposed that all infants are biologically disposed to seek and maintain proximity to the caregivers in the face of potential or real threat (63) but that there are individual differences in how this is achieved and expressed behaviourally. Only the validated assessments of attachments should be used to assess this (2, +3) and observing parent-child separations are key to this (9, +5). Not just assessments of child attachment, but the adult attachment interview too can be also be helpful for understanding the needs and challenges of parents of children in services (8, +3).

Some areas of attachment research remain unknown or haven’t been very convincing. For instance, we really don’t know to what extent early attachment experiences impact on brain development (51, 0), though lots of people have tried writing about this. We also don’t really know if childhood attachment patterns are good predictors of adult relationship functioning (24, 0). However, I want to be very clear about the attachment disorganisation classification. It should not be used to identify child maltreatment (1, −4) and is absolutely not an inevitable consequence of trauma, abuse or neglect (62, −5). In fact, it is possible to have a secure relationship with a maltreating caregiver (21). It is also important to remember that attachment disorders are rare (28).

I can’t be very specific about what is helpful about attachment theory for clinical practice. For example, I don’t know how it plays out or should play out in key tasks such as differential diagnosis (42, 1) or treatment planning. I also don’t know what sorts of attachment problems are most prevalent in services (23, 0), or how much attention should be given to attachment relative to other theories of child development (15, −1). I am unsure whether attention problems are related to attachment (40, −1) or how attachment problems should be prioritised over other concerns (36, −1).

### Autodidactic, therapeutic, and enthusiastic perspective

The more I work in child services the more I have learnt about attachment and realise how relevant it is to our understanding of children. Above all, insecure attachment is big problem for child mental health (27, −5), perhaps especially for children of parents with learning disabilities (29). Yet this big problem is not adequately recognised since attachment theory isn’t given enough focus and attention to in services (15, −4). Part of working in an attachment-informed way means providing a safe environment for children to explore (64, +3). As well as seeking to contribute to children’s sense of access to a secure base and safe haven, we should also use the adult attachment interview (8, +4) and use interventions that target parents’ internal working models of attachment (46, +3).

I have to work really hard to help my colleagues understand (17) and think about attachment. I mostly learned about it through recent psychotherapeutic literature rather than Bowlby’s original books. I think that it is very relevant (13) to the families we see. All behaviour is attachment behaviour really (49, −3). We need to be able to assess it properly (5, −2) as addressing attachment insecurity is a clinical priority (36, +2). Early attachment experiences determine how the brain develops (51, +5), tell us about how children will get on in adolescence (20) and how they will function in their adult relationships (24, +2). But that doesn’t mean it is too late; attachment interventions can work for adolescents as well as children (58, +2). Not everyone needs an attachment-informed intervention (60, +1), but for those who do it is really important, so I have taken it upon myself to learn more.

Childhood trauma, abuse and neglect prevent children from developing secure attachments (31, +2) and lead to huge problems. Some children’s behaviour shows that they don’t always want to be close to their attachment figures when they are ill or frightened (63, −1) and we have to work with them very carefully to re-establish this. It is not appropriate to use short interventions for these children (10, −4) as they need longer term work. You could call such severe attachment problems disorganised, but I don’t like to use that term (23, −3). I don’t believe attachment can really be disordered as children ultimately adapt to their caregiving environment (34, +5) so I’m not keen on that diagnosis either and I don’t really know what it means (43, −2).
